# Transcriptome Profile in the Mouse Brain of Hepatic Encephalopathy and Alzheimer’s Disease

**DOI:** 10.3390/ijms24010675

**Published:** 2022-12-30

**Authors:** Young-Kook Kim, Yoon Seok Jung, Juhyun Song

**Affiliations:** 1 Department of Biochemistry, Chonnam National University Medical School, Hwasun 58128, Jeollanam-do, Republic of Korea; 2 Department of Anatomy, Chonnam National University Medical School, Hwasun 58128, Jeollanam-do, Republic of Korea

**Keywords:** hepatic encephalopathy (HE), Alzheimer’s disease (AD), bile duct ligation (BDL) model, 5×FAD model, RNA sequencing

## Abstract

Hepatic encephalopathy (HE) is a chronic metabolic disease accompanied by neuropathological and neuropsychiatric features, including memory deficits, psychomotor dysfunction, depression, and anxiety. Alzheimer’s disease (AD), the most common neurodegenerative disease, is characterized by tau hyperphosphorylation, excessive amyloid beta (Aβ) accumulation, the formation of fibrillary tangles, hippocampus atrophy, and neuroinflammation. Recent studies have suggested a positive correlation between HE and AD. Some studies reported that an impaired cholesterol pathway, abnormal bile acid secretion, excessive ammonia level, impaired Aβ clearance, astrocytic dysfunction, and abnormal γ-aminobutyric acid GABAergic neuronal signaling in HE may also be involved in AD pathology. However, the mechanisms and related genes involved in AD-like pathology in the HE brain are unclear. Thus, we compared the cortical transcriptome profile between an HE mouse model, bile duct ligation (BDL), and an AD mouse model, the 5×FAD. Our study showed that the expression of many genes implicated in HE is associated with neuronal dysfunction in AD mice. We found changes in various protein-coding RNAs, implicated in synapses, neurogenesis, neuron projection, neuron differentiation, and neurite outgrowth, and non-coding RNAs possibly associated with neuropathology. Our data provide an important resource for further studies to elucidate AD-like pathophysiology in HE patients.

## 1. Introduction

Hepatic encephalopathy (HE) is a neuropsychiatric disorder caused by both acute and chronic liver failure and is accompanied by cognitive impairment and brain dysfunction leading to coma [1,2,3]. HE is also characterized by depressive mood changes, personality changes, anxiety, attention deficits, and abnormal motor function [1,4]. Among HE types, overt HE occurs in around 40% of patients with liver cirrhosis, and minimal HE occurs in approximately 80% of patients with liver cirrhosis [1,4,5]. Although the pathogenesis and mechanisms of HE brain dysfunction are still unknown, many researchers suggest that the excessive production of ammonia in the liver is involved [6,7]. These alterations of the metabolism in HE contribute to neuroinflammation, impaired brain energy metabolism, and blood–brain barrier (BBB) disruption [6,7]. Some studies have shown that HE brains display microglia activation, M1 phenotype microglia induction, and astrocyte dysfunction leading to severe neuroinflammation [8,9]. In HE brains, reactivated astrocytes lead to an imbalanced glutamate metabolism and impaired energy metabolism under high ammonia conditions, leading to neuronal dysfunction [9].

The bile duct ligation (BDL) model, which involves the double ligation of the common bile duct without transection [10], is a hepatotoxin model widely used to study HE pathology caused by acute liver failure [11,12,13]. The BDL model is characterized by dilation of the gall bladder, cholestasis, liver portal inflammation, hepatocyte necrosis and apoptosis, and liver fibrosis [14,15,16].

Alzheimer’s disease (AD) is the most common type of dementia, affecting more than 40 million people worldwide, and is one of the main causes of mortality [17]. The risk factors of AD include genetic factors, metabolic imbalance, impaired blood circulation, abnormal lipid profile, impaired energy metabolism, insulin resistance, and inflammation [18]. Diverse metabolic syndromes, including obesity, diabetes, and dyslipidemia, are strongly linked to the onset and development of AD [19,20].

Interestingly, many researchers have suggested that an abnormal level of alanine aminotransferase (ALT) and aspartate aminotransferase (AST), used to measure liver function [21], contributes to the onset and development of AD and cognitive deficits [22,23,24]. Other studies mentioned that liver failure, such as nonalcoholic fatty liver disease (NAFLD) and chronic Hepatitis C viral infection, is positively correlated with dementia, such as AD [25,26]. Although there are considerable commonalities between liver failure and AD pathogenesis, the relationship, and mechanisms between the two diseases are not fully understood.

Thus, we compared the transcriptomes between the brain cortex of an HE BDL mouse model and the brain cortex of an AD 5×FAD mouse model. We investigated the alteration of diverse protein-coding RNAs and long non-coding RNAs (lncRNAs) in the cortex of BDL and 5×FAD mouse models. We identified that the functions of commonly changed RNAs are related to synaptic function in AD and HE brains. Our data might be a critical resource to understand AD-like neurological problems in HE pathogenesis.

## 2. Results

**Figure 1:**
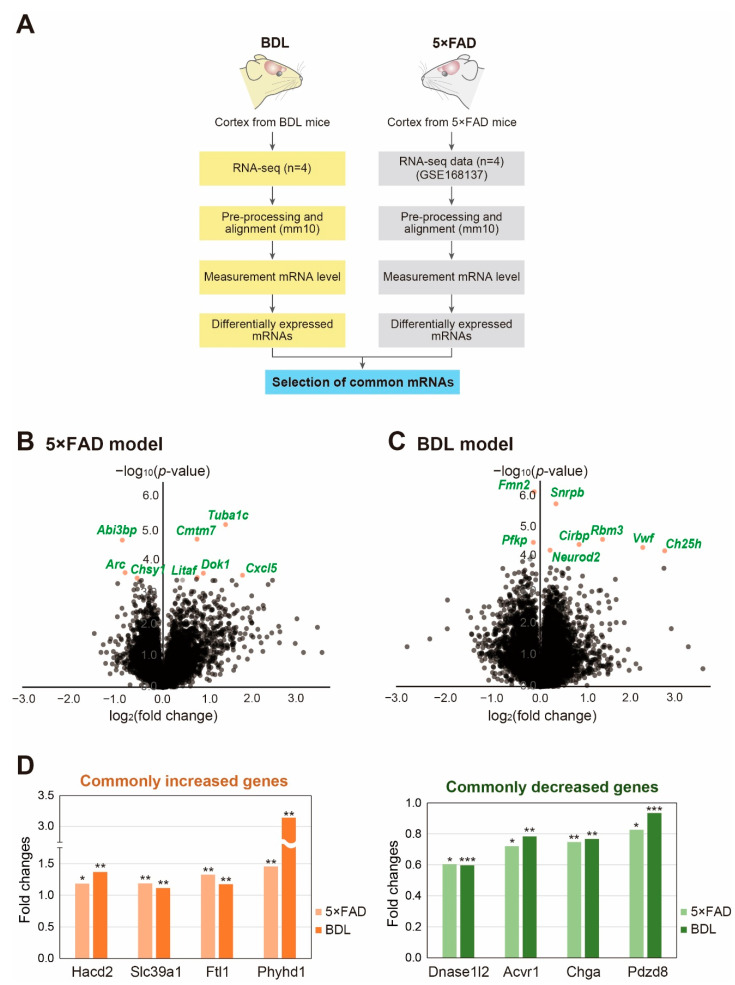
**Analysis of transcriptomic data from the cortex from BDL and 5×FAD mouse models:** (**A**) flowchart showing the process for analyzing the transcriptome data; (**B**,**C**) Volcano plots of the (**B**) 5×FAD group and (**C**) BDL group. The X-axis represents the log2-transformed fold change in each group and the Y-axis represents the −log_10_(*p*-value). Red dots depict the significantly changed genes; and (**D**) the common genes with a significant expression change between the cortex of BDL and 5×FAD models. The graphs depict commonly increased and decreased genes. An unpaired two-tailed *t*-test was used for the statistical analysis. * *p* < 0.05, ** *p* < 0.01, *** *p* < 0.001.

For transcriptome analysis of the 5×FAD and BDL brain cortices, we conducted RNA sequencing of total RNA in the cortices of the BDL model and corresponding control mice. For the RNA sequencing data of the 5×FAD mouse, we used the publicly available dataset from the GEO database (GSE168137). After analyzing the RNA sequencing data (Figure 1A), the genes with high expression and statistically significant changes in each group were presented as volcano plots (Figure 1B,C). In the 5×FAD mouse brain cortices (Figure 1B), there were 480 genes with significantly increased expression and 486 genes with significantly decreased expression (*p* < 0.05). As depicted in the volcano plot, the expression of tubulin alpha 1c (*Tuba1c*), CKLF-like MARVEL transmembrane domain containing 7 (*Cmtm7*), ABI family member 3 binding protein (*Abi3bp*), activity-regulated cytoskeleton-associated protein (Arc), chondroitin sulfate synthase 1 (*Chsy1*), lipopolysaccharide-induced TNF factor (*Litaf*), docking protein 1 (*Dok1*), and C-X-C motif chemokine ligand 5 (*Cxcl5*) were significantly differentially expressed between the control and 5×FAD mouse brain cortices (Figure 1B). In the BDL model, there were 916 genes with significantly increased expression and 897 genes with significantly reduced expression (*p* < 0.05) (Figure 1C). As depicted in the volcano plot, formin 2 (*Fmn2*), small nuclear ribonucleoprotein polypeptides B and B1 (*Snrpb*), Von Willebrand factor *(Vwf)*, cold-inducible RNA-binding protein (*Cirbp*), RNA-binding motif protein 3 (Rbm3), cholesterol 25-hydroxylase (*Ch25h*), neuronal differentiation 2 (*Neurod2*), and platelet-type phosphofructokinase (*Pfkp*) were significantly differentially expressed between the sham control cortices and BDL mouse cortices (Figure 1C). To identify genes whose expression changes in both 5×FAD and BDL models, we selected 500 genes from each group with the lowest *p*-values. Four genes, including 3-hydroxyacyl-CoA dehydratase 2 (*Hacd2*), solute carrier family 39 member 1 (*Slc39a1*), ferritin light polypeptide 1 (*Ftl1*), and phytanoyl-CoA dioxygenase domain-containing 1 (*Phyhd1*), were found to be increased in both the 5×FAD and BDL models (Figure 1D), whereas four genes, including deoxyribonuclease 1-like 2 (*Dnase1.2*), activin A receptor type I (*Acvr1*), chromogranin A (*Chga*), and PDZ domain-containing protein 8 (*Pdzd8*), were decreased in both models (Figure 1D).

**Figure 2:**
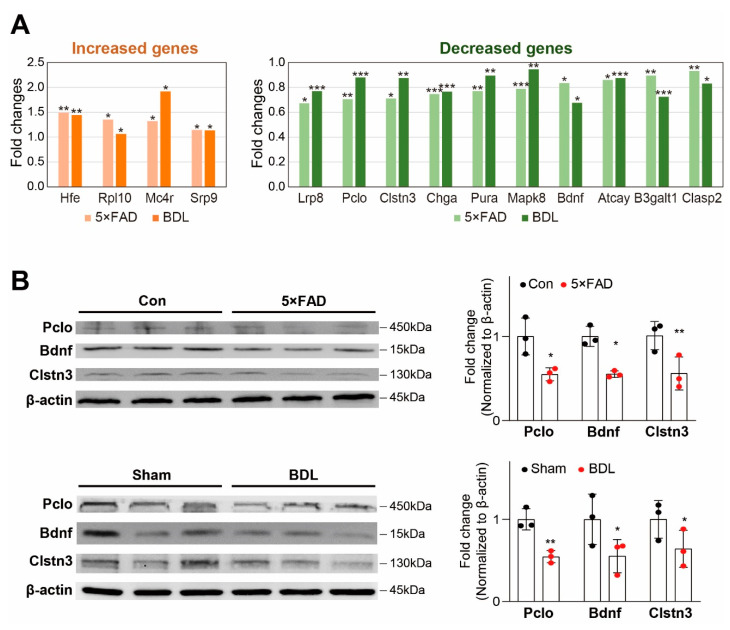
**Selected significantly differentially expressed genes in the mouse cortex of BDL and 5×FAD models:** (**A**) the significantly changed genes related to neuropathology are shown. The graphs depict four genes increased in both models (**left**) and ten genes decreased in both models (**right**). An unpaired two-tailed *t*-test was used for the statistical analysis. * *p* < 0.1, ** *p* < 0.05, *** *p* < 0.01; (**B**) the measurement of Pclo, Bdnf, and Clstn3 protein levels in the cortex of the 5×FAD and BDL models. Data are represented as mean ± standard error of the mean (S.E.M) (*n* = 3). An unpaired two-tailed *t*-test was used for the statistical analysis. * *p* < 0.05, ** *p* < 0.01.

Next, we examined the expression of commonly changed genes associated with neuropathology (Figure 2A). We first selected the increased or decreased genes with a *p*-value of 0.1 or less in both the 5×FAD and BDL brain cortices. By manually curating the list of genes based on the literature, we chose the protein-coding genes associated with neurological diseases and selected those genes related to neuropathology observed during the progression of AD and HE (Figure 2A). Our analysis showed that the genes related to neuropathology that were increased in both models were homeostatic iron regulator *(Hfe)*, ribosomal protein L 10 *(Rp110)*, melanocortin-4 receptor *(Mc4r)*, and signal recognition particle 9 *(Srp9)*, and those decreased in both models were low-density lipoprotein receptor-related protein 8 *(Lrp8)*, piccolo *(Pclo)*, calsyntenin 3 *(Clstn3)*, chromogranin *(Chga)*, purine-rich element-binding protein A *(Pura)*, MAPK8 mitogen-activated protein kinase 8 *(Map8)*, brain-derived neurotrophic factor *(Bdnf)*, 3-beta-glucuronosyltransferase 1 *(B3galt1)*, ATCAY kinesin light chain-interacting caytaxin *(Atcay)*, and cytoplasmic linker-associated protein 2 *(Clasp2)* (Figure 2A).

Among the genes significantly differentially expressed, we confirmed the protein expression of synapse-related *Pclo*, *Bdnf*, and *Clstn3* genes in both the 5×FAD and BDL cortices (Figure 2B). To confirm the protein expression and compare their levels between 5×FAD and BDL cortices, western blotting was performed using mice from both models and two controls (normal male BDL sham control). Our results showed that the protein levels of Pclo, Bdnf, and Clstn3 were significantly decreased in the cortices of both the 5×FAD and BDL mice (Figure 2B). Since these genes have previously been reported to be involved in neuropathology, such as synaptic plasticity, our data suggest that the change in expression may be important in the cerebral cortex of AD and HE brains.

**Figure 3:**
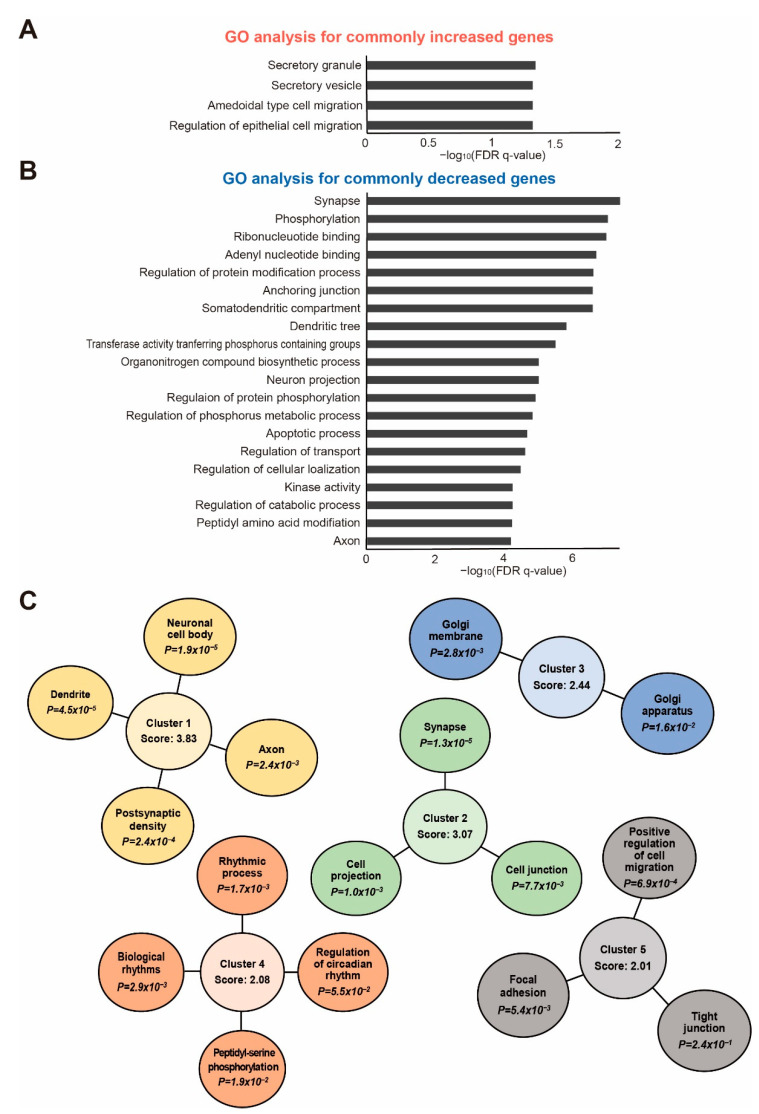
**Functional analysis of the differentially expressed genes common to both BDL and 5×FAD models:** (**A**) GO analysis for the commonly increased genes. The top four GO terms based on false discovery rate (FDR) q-values are shown; (**B**) GO analysis for the commonly decreased genes. The top 20 GO terms based on false discovery rate (FDR) q-values are shown; and (**C**) the functional annotation clustering based on the DAVID analysis tool. The top five clusters with a significant change are shown.

Next, we performed a GO analysis in MSigDB to identify cellular pathways associated with the differentially expressed genes common in the brain cortex of both the 5×FAD and BDL models (Figure 3A,B). For this analysis, differentially expressed genes with a *p*-value of 0.1 or less in both groups and whose expression increases and decreases similarly were selected. Through this process, a list of 51 genes that were increased in both models, and 130 genes that were decreased in both models was obtained. The GO analysis of the increased genes revealed that GO pathways related to secretory granules, secretory vesicles, ameboidal type cell migration, and regulation of epithelial cell migration were most significantly enriched (Figure 3A). The GO analysis of the decreased genes showed that the GO pathways related to synapses, phosphorylation, ribonucleotide binding, adenyl nucleotide binding, and regulation of protein modification processes were most significantly affected (Figure 3B).

Additionally, we performed a functional clustering analysis of the increased genes using the DAVID functional annotation tool [27]. For this analysis, we used genes most differentially expressed in both the 5×FAD and BDL models. We observed five highly enriched clusters, and the two most highly enriched clusters were related to neuronal function, including dendrite, axon, and synapse (Figure 3C).

**Figure 4:**
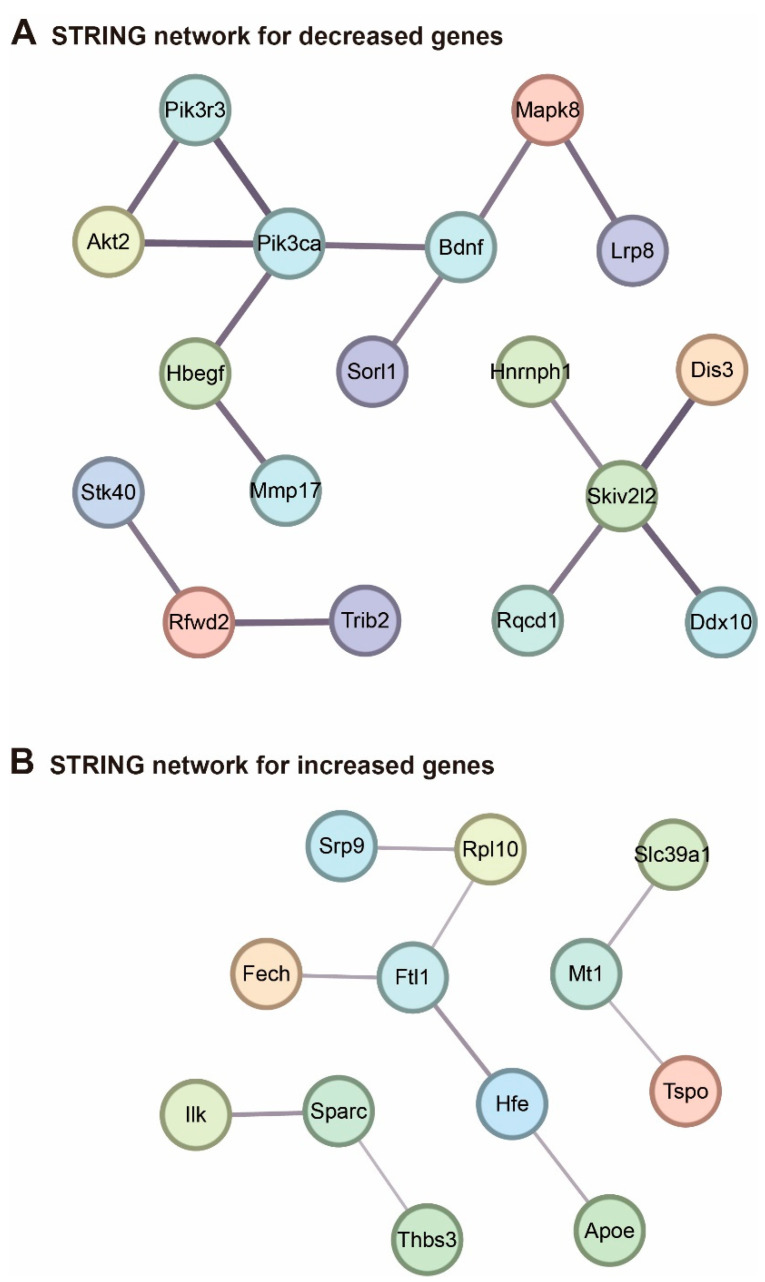
Analysis of protein interaction network for differentially expressed genes common to the BDL and 5×FAD models using STRING database: (**A**) protein–protein interaction network for genes decreased in both models. Those networks with minimum nodes greater than two were selected. The minimum score was set as 0.7 (high confidence); and (**B**) protein–protein interaction network for genes decreased in both models. Those networks with minimum nodes greater than two were selected. The minimum score was set as 0.4 (medium confidence).

To analyze protein networks affected in the two models similarly, we utilized the significantly selected genes above and applied them to the STRING network analysis database [28] (Figure 4). The protein interaction network obtained from the STRING database for the genes decreased in both models is shown in Figure 4A, while that for the genes increased in both models is depicted in Figure 4B. Interestingly, Bdnf is shown as part of the network containing gene decreased in both models (Figure 4A). This result suggests that the signaling pathway involving Bdnf-mediated regulation may have a common role in the cortex of 5×FAD and BDL models.

**Figure 5:**
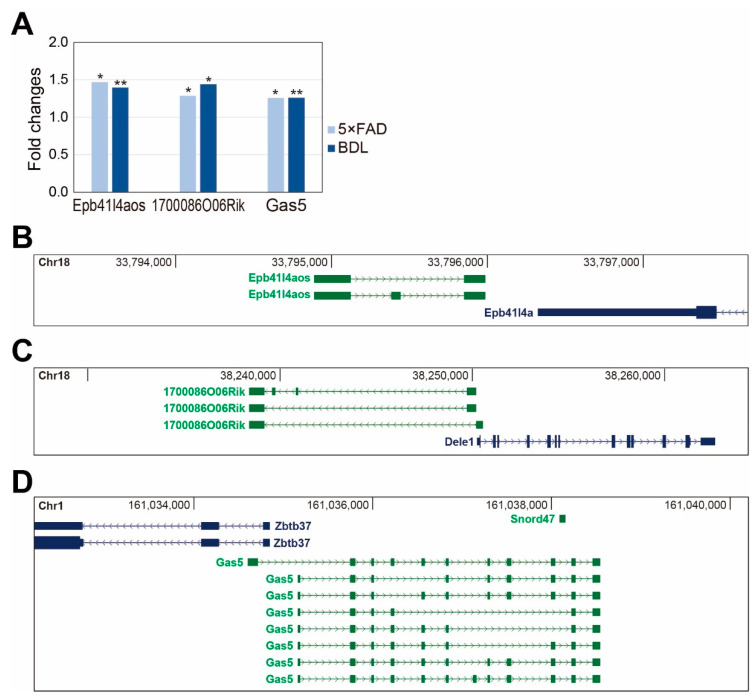
**The differentially expressed lncRNAs common to both BDL and 5×FAD models:** (**A**) the top three lncRNAs based on the common expression changes were selected and shown. An unpaired two-tailed *t*-test was used for the statistical analysis. * *p* < 0.1, ** *p* < 0.01; and (**B**–**D**) among the selected lncRNAs (**A**), those lncRNAs with neighboring protein-coding genes based on the genomic locus were selected. The genomic information for (**B**) Epb41l4aos, (**C**) 1700086O06Rik, and (**D**) Gas5 were obtained and modified from The Genome Browser.

Finally, we screened candidate long non-coding RNAs (lncRNAs) that were differentially expressed in the cortex of both models. LncRNAs are a group of non-coding RNAs longer than 200 nucleotides and have been reported to be involved in the development of neuropathogenesis in many studies [29]. We discovered three common lncRNAs, including Epb41l4aos, 1700086O06Rik, and Gas5, which were significantly altered in both cerebral cortices (Figure 5A). Epb41l4aos is located on chromosome 18 in the antisense strand against the Epb41l4a gene at the genomic locus (Figure 5B). 1700086O06Rik is located on chromosome 18 near the Dele1 gene at the locus (Figure 5C), and Gas5 is located on chromosome 1 with Zbtb37 as its neighboring gene (Figure 5D). Since many lncRNAs regulate the expression of neighboring genes, and Dele1 and Zbtb37 have been reported to be involved in neuronal processes, we hypothesize that the lncRNAs, 1700086O06Rik and Gas5, also have important functions in HE and AD cortices.

## 3. Discussion

Here, we investigated transcriptional commonalities and differences in models for AD and HE, the 5×FAD and BDL cortices, respectively. First, we analyzed genes significantly differentially expressed in each group. We observed distinguished expression of several genes, such as *Tuba1c*, *Dok1*, and *Cxcl5*, in the 5×FAD cortex (Figure 1B). *Tuba1c* is an α-tubulin subtype known to be highly expressed in glioma brains [30] and is related to immune cell infiltration in the brain [31], and cell mitosis and division [32]. *Dok1* is associated with TLR4 inflammatory signaling [33], Ras-extracellular signal-regulated kinase (ERK) signaling [34], CD200 receptor immune signaling [35], and TLR2 inflammatory signaling in glia [36]. *Cxcl5* expression is dramatically increased in AD brains and contributes to severe neuroinflammation [37]. One study demonstrated that reduced *Arc* could reduce the risk for AD [38] and reduce synaptic plasticity [39]. Thus, the 5×FAD mouse cortex exhibits the alteration of synaptic plasticity-, glioma-, and neuroinflammation-related genes.

In addition, we found distinguished expression of other genes, including *Snrpb*, *Rbm3*, *Ch25h*, *Vwf*, *Neurod2*, and *Fmn2* in the BDL cortex (Figure 1C). *Snrpb* is associated with glioblastoma [40], and *Rbm3* is involved in neuronal activity, neurogenesis, and synaptic vesicle dynamics in damaged brains [41,42]. *Neurod2* is positively associated with synapse formation, synaptic density protein level, and dendritic spine maturation, leading to the onset of neurological diseases [43,44]. *Vwf* is related to neuroinflammatory responses and promotes permeability and disruption of the BBB in the brain [45]. Additionally, higher *Vwf* gene expression has been observed in neurological diseases, such as stroke and venous sinus thrombosis [46]. BBB disruption is a critical factor in promoting the progression of AD [47]. *Ch25h* is a susceptibility gene for the onset of AD [48] and deepens chronic neuroinflammation by activating the NLRP3 inflammasome [49]. *Fmn2* is related to cell cycle arrest, DNA damage against stress conditions [50], and the pathogenesis of neuropsychiatric disorders and dementia [51]. Taken together, the BDL HE model shows changes in synapse formation-related genes, neurovascular dysfunction, and neuroinflammation in the brain cortex, which can ultimately lead to memory impairment, one of the neuropathological features observed in AD.

Considering that the HE brain has been previously shown to exhibit AD-like pathological alterations, including impaired synaptic transmission and memory deficits [52], our analysis elucidates gene expression changes that may be correlated with the common pathologies. The genes increased in both models in this current study were *Hacd2*, *Slc39a1*, *Ftl1*, and *Phyhd1* (Figure 1D). A previous study showed that *Hacd2* is involved in the fatty acid elongation pathway [53]. *Slc39a1* is a zinc ion transport protein related to the progression of glioma by promoting MMP2 and MMP9 [54], and is increased in the progression of schizophrenia [55]. Increased expression of *Ftl1* gene has been observed in patients with neuroferritinopathy [56,57] and causes iron metabolism dysregulation, which affects the pathogenesis of the neurodegenerative disease, such as AD [58].

The genes decreased in both models were *Dnase1.2*, *Acvr1*, *Chga*, and *Pdzd8*. *Dnase1* gene is related to DNA repairing, and Dnase I is used to treat AD neuropathologies [59], cystic fibrosis [60], and cancer [61]. *Acvr1* is changed in patients with fibrodysplasia ossificans progressiva, accompanied by cognitive decline, sensory abnormality, and cerebellar abnormality [62,63,64]. *Acvr1* is also involved in neurogenesis and hippocampal volume size [65]. Decreased expression of *Pdzd8* exacerbates the imbalance in mitochondrial homeostasis and neuronal dysfunction and increases Aβ_42_ toxicity in the brain [66]. These data suggest that 5×FAD and BDL mouse cortices show genetic alterations commonly associated with neuronal dysfunction, iron metabolism dysregulation, neurovascular dysfunction, reduced neurogenesis, increased inflammatory responses, mitochondrial dysfunction, and cognitive impairment.

We also showed four genes related to neuropathology were increased in both the AD and HE models, including *Hfe*, *Rp110*, *Mc4r*, and *Srp9*. *Hfe* is related to iron accumulation-induced memory deficits in AD pathology [67]. *Mc4r* has been shown to suppress hippocampal synaptic plasticity and long-term potentiation in AD brains [68]. *Srp9* impairs the expression of α-amino-3-hydroxy-5-methyl-4-isoxazole propionic acid (AMPA) and N-methyl-D-aspartate (NMDA) receptors in hippocampal neurons, which is involved in neurological diseases, such as seizures [69].

Among the ten genes related to neuropathology, we found to be decreased in both AD and HE models, *Lrp8* has been shown to be related to increased risk for AD [70]. In addition, *Pura* has been known to exert a neuroprotective effect in neurodegenerative disease brains [71]. *Pclo* and *Bdnf* are related to stable synapse transmission [72] and neuronal synaptic function [73], respectively. Also, *Clasp2* is associated with microtubule stabilization in neuronal cell differentiation and axon elongation [74].

In addition to our data on neuropathology-related genes, genes related to synaptic plasticity were also found to be decreased in both AD and HE cortices, including *Pclo*, *Bdnf*, and *Clstn3. Pclo* is associated with presynaptic cytomatrix protein, and reduced *Pclo* expression leads to neuropsychiatry diseases, such as bipolar disorder and major depressive disorder [72,75]. *Bdnf* is well known to be linked to neurogenesis and synaptic plasticity and is involved in the development of dementia [73]. Bdnf levels in the cerebrospinal fluid of AD patients are reduced, and decreased *Bdnf* expression affects brain dysfunction and temporal lobe atrophy [76]. In addition, *Clstn3* has been shown to lead to AD by regulating Aβ accumulation and neurite formation in the brain [77,78].

The GO data for the genes increased in both 5×FAD and BDL mouse cortices showed altered cellular signaling associated with secretory granules, secretory vesicles, and epithelial cell migration. Extracellular vesicles are vesicles originating from different intracellular compartments [79] and are sorted into exosomes, apoptotic bodies, and microvesicles [79]. Several studies have shown that extracellular vesicle secretion is important for removing toxic proteins, such as tau [80] and Aβ plaques [81,82], in the cerebrospinal fluid of AD patients [83] and for regulating inflammatory responses [84]. An impaired extracellular secretory vesicle system results in neuronal cell death, loss of BBB integrity, and synaptic dysfunction leading to cognitive decline [85,86,87]. Excessive toxic protein accumulation in the brain may accelerate the extracellular secretory vesicle system.

The GO data for the genes decreased in both 5×FAD and BDL mouse cortices showed changed cellular signaling related to synapses, dendritic tree, neuron projection, metabolic processes, transport regulation, apoptotic processes, and axons, and are involved in AD pathologies. Furthermore, the DAVID clustering data showed that common genes in both groups were closely associated with postsynaptic density, axon, dendrite, synapse, cell projection, biological rhythms, and tight junction (Figure 3C). Numerous previous studies have reported that synaptic dysfunction related to impaired synaptic transmission is observed in AD [88] and HE brains [89]. Some studies have reported that synapse loss and synaptic dysfunction are key features of AD brains [90] and could be used as indicators of the predicted stage of AD development [91,92]. Synaptic dysfunction leads to cognitive impairment by inhibiting spine maturation and aggravating the development of AD [93,94,95,96].

Axonal degradation, impaired axonal transport, and poor neuronal projection weaken axonal connectivity and synaptic function between diverse brain regions [97]. AD brains exhibit axonal degeneration [98], a poor axonal tract in the hippocampus [99], abnormal axonal extension, and loss of synaptic connectivity between the medial temporal lobe and cortical areas [100]. AD brains have been shown to exhibit neuronal and glial cell death via apoptosis and autophagy, leading to neuroinflammation and memory deficits [101,102]. In the brain, excitatory synapses create dendritic spines for electric neuronal signaling connections involved in memory formation [103]. Spine immaturity, loss of postsynaptic density proteins, and loss of presynaptic elements are major features in AD brains [104]. Therefore, we suggest that both HE and AD brains exhibit impaired synaptic plasticity, synaptic transmission, poor neurite outgrowth, increased apoptotic processes, tight junction protein loss, and postsynaptic density loss. We emphasize that these changes can eventually lead to cognitive deficits.

In addition, our protein network analysis suggests that the same diverse pathways might be changed in both disease models (Figure 4). According to the STRING network analysis for the genes decreased in both models (Figure 4A), *Pik3ca*, a gene related to neuronal hyperactivity [105], interacts with *Bdnf*, a neurotrophic factor [106]. *Pik3ca* also interacts with *Lrp8*, a gene related to the onset of neuropsychiatric diseases, such as schizophrenia [107], *Mapk8*, a gene associated with reduced apoptosis in glioblastoma cells [108], and *Sorl1*, which is decreased in AD brains by regulating Aβ accumulation [109]. Overall, increased expression of protein-coding genes in both models is associated with reduced neurotrophic factor production, impaired lipoprotein density proteins, decreased cell survival response, and impaired Aβ clearance in the brain.

Furthermore, the STRING network analysis for genes increased in both models (Figure 4B) showed that *Ftl1*, involved in neuroferritinopathy [110], interacts with *Rpl10*, a gene linked with autism progression [111], *Srp9*, a gene related to seizure susceptibility [69], and *Apoe*, a gene related to axon demyelination and impaired synapse formation in AD [112]. Also, *Tspo*, a gene related to neuronal damage and increased inflammation [113] interacts with *Slc39a1*, a regulator of immune responses and tumor malignant progression [114]. In addition, *Sparc*, related to immune cell migration and Aβ protein deposition [115], interacts with *Ilk*, a gene linked with glioma cell migration [116]. These data suggest that protein-coding genes increased in both models are linked to the increase of immune responses and inflammation, impaired iron metabolism, and Aβ accumulation in the brain.

We also showed that lncRNAs, including Epb41l4aos, 1700086O06Rik, and Gas5, showed significant changes in both groups (Figure 5A). Epb41l4aos exists on chromosome 18 and is located in the antisense strand near the *Epb41l4a* gene (Figure 5B). 1700086O06Rik exists on chromosome 18 and is located near the *Dele1* gene (Figure 5C). Gas5 exists on chromosome 1 and is located near the *Zbtb37* gene (Figure 5D). A previous study demonstrated that lncRNAs could control neighboring genes through transcriptional and posttranscriptional processing [117]. One recent study reported that *Dele1* could boost mitochondrial dysfunction and cell death signaling against stress conditions [118]. However, the expression of *Dele1* mRNA was not changed in our models (data not shown). Furthermore, Gas5 exists on chromosome 1 and is located near the *Zbtb37* gene (Figure 5D). One study reported that Gas5 deactivates anti-inflammatory phenotype M2 polarization in microglia and aggravates the demyelination process in neurons, leading to memory deficits [119]. Another study reported that Gas5 suppresses neuronal differentiation and function in brain injury conditions [120]. However, the expression level of *Zbtb37* was not changed in either disease model in our study (data not shown). Thus, the working mechanism of the selected lncRNAs in our study might not be related to the regulation of neighboring genes in our model, and further studies are required to identify their regulatory mechanism.

In conclusion, our transcriptome analysis data showed protein-coding genes and lncRNAs in differentially expressed in both HE and AD brain cortices that are considerably associated with AD-like neuropathology, such as synaptic dysfunction, impaired axon elongation, increased inflammatory responses, Aβ accumulation, neurovascular dysfunction, and memory deficits. Since our data focus on the cortex in HE and AD mouse models, further studies are needed to understand the differences and similarities between HE and AD in other brain regions. Therefore, we suggest that further studies comparing HE and AD brains are essential for finding specific genetic connections and molecular mechanisms for clinical treatment solutions.

## 4. Materials and Methods

### 4.1. Preparation of Animals for BDL Surgery

To investigate the transcriptome from the cortex of BDL mice, we purchased C57BL/6 male mice aged 12 weeks from Orient Bio (Seongnam, Gyeonggi-Do, Republic of Korea) for this study. These animals were provided free access to food and water for the study duration following Chonnam National University animal center ethics. The mice were anesthetized in mixed gas and maintained with 2% isoflurane during the bile duct ligation surgery. The skin of the mice was sterilized with 70% ethanol, and the abdomen was dissected using surgical scissors. After the abdomen opening, the bile duct was ligated using a black silk suture. After ligation, the peritoneum and abdominal skin were sutured with black silk suture thread and sterilized with 70% ethanol. Mice were housed in their home cages and then sacrificed two weeks later. Mouse brains were isolated after perfusion with saline, and the cortex was separated and kept at −70 °C until use. The experiments were conducted following the recommendations from “96 Guidance for Animal Experiments” established by the Animal Ethics Committee at Chonnam National University. The study was conducted following the ARRIVE guidelines.

### 4.2. Preparation of 5×FAD Brain Cortex

Five-month-old male 5×FAD transgenic mice (strain B6SJL-Tg [APPSwFlLon,PSEN1*M146L*L286V]6799Vas/J) were obtained from The Jackson Laboratory (Bar Harbor, ME, USA). Wild-type male C57BL/6 mice (260–310 g, 5 months) were obtained from Orient (Pyeongtaek, Republic of Korea). Aβ_42_ accumulation was assessed in all brain tissue from animals at two months of age. The experiment was performed following the recommendations of “96 Guidance for Animal Experiments,” established by the Animal Ethics Committee at Chonnam National University. We isolated the cortical tissue from three 5×FAD mice and three wild-type mice for conducting several experiments.

### 4.3. RNA Sequencing

Total RNA from the cerebral cortex in control and BDL mice (*n* = 3 each) was extracted using TRIzol reagent (Thermo Fisher, Waltham, MA, USA), and the integrity was checked using the Agilent 2100 BioAnalyzer (Agilent, Santa Clara, CA, USA). Total RNA was treated with a Ribo-Zero Gold rRNA Removal Kit (Illumina, San Diego, CA, USA) to remove ribosomal RNA, and RNA sequencing libraries were prepared using a TruSeq Stranded Total RNA Kit (Illumina). The RNA libraries were paired-end sequenced with 100 sequencing cycles on a HiSeq 2500 system (Illumina).

### 4.4. The Data Used to Analyze the Transcriptome of the 5×FAD Mouse

To compare the common transcriptomic profile between BDL mice and 5×FAD mice cortices, we obtained RNA sequencing data from the cerebral cortices of three 8-month male 5×FAD mice from the Gene Expression Omnibus (GEO) database (accession number GSE168137) [121]. Each group contained the data from five mice (*n* = 5).

### 4.5. Analysis of RNA Sequencing Data

Among the sequencing data produced from BDL and 5×FAD models, those with low-quality sequencing reads were trimmed using Trimmomatic [122] (Figure 1A). The trimmed sequences were aligned to the mouse genome (mm10) by using the spliced transcripts alignment to a reference (STAR) aligner [123]. Cuffnorm was also used to estimate normalized values of fragments per kilobase of transcript per million mapped reads (FPKM) based on GENCODE annotation (Release M17, GRCm38.p6 [124]) (Figure 1A). Transcripts with an average FPKM value of less than 1 or transcripts not detected in any sample were deleted from additional analysis (Figure 1A).

### 4.6. Functional Analysis of Differentially Expressed Genes

To select the differentially expressed genes common to the BDL and 5×FAD models, we first selected transcripts with a significantly different expression between the disease group and its corresponding control group in the BDL and 5×FAD data. For this, the top 500 genes with significant expression change based on *p*-value were selected in each of the BDL and 5×FAD model datasets. Among them, the genes differentially expressed the same in the BDL and 5×FAD models were selected. This filtering resulted in four genes that were decreased and four genes that were increased in both models. For gene ontology (GO) analysis using the Molecular Signatures Database [125], we selected the genes with *p*-values less than 0.1 in both models. For the same group of genes, functional annotation clustering was performed using the Database for Annotation, Visualization and Integrated Discovery (DAVID) tool [27]. The STRING (http://string-db.org (accessed on 3 November 2022)) software program was used to find the interaction network among the selected genes. Only the networks with minimum nodes greater than two were selected.

### 4.7. Western Blot Analysis

The tissues were lysed in ice-cold radioimmunoprecipitation assay (RIPA) buffer (Translab, Sacramento, CA, USA) for 20 min on ice. We used a bicinchoninic acid (BCA) protein assay kit (Thermo Fisher, Waltham, MA, USA) to assess the protein concentration of the protein extract. Protein (70 μg) was separated on 12% SDS-PAGE, and then transferred onto PVDF (Millipore) activated by absolute methanol. The PVDF membrane was blocked with 5% skim milk (BD Bioscience, San Diego, CA, USA) in 1X TBS-T buffer for one hour at room temperature. Membranes were incubated with primary antibodies overnight at 4 °C. Primary antibodies used are as follows: Pclo (Abcam, ab20664; diluted 1:1000), Clstn3 (Proteintech, 13302-1-AP; diluted 1:1000), Bdnf (Abcam, ab108319; diluted 1:1000), and β-actin (AbFrontier diluted 1:5000). After primary antibody incubation, the membranes were incubated with horseradish peroxidase (HRP)-labeled secondary antibody (1:5000 dilution) for two hours at room temperature. The protein bands were detected using an ECL solution (Thermo Fisher Scientific) and iBright CL1000 imaging system (Invitrogen) according to the manufacturer’s instructions. Protein levels were normalized to β-actin protein levels.

### 4.8. Statistical Analysis

We analyzed the data using unpaired two-tailed *t*-tests with Welch’s correction between groups. Data were considered significant at * *p* <0.05, ** *p* < 0.01, and *** *p* < 0.005.

## Data Availability

The data presented in this study are available on request from the corresponding author.

## Abbreviations

Hepatic encephalopathy (HE), Alzheimer’s disease (AD), γ-aminobutyric acid (GABA), Blood-brain barrier (BBB), Bile duct ligation (BDL), Alanine aminotransferase (ALT), Aspartate aminotransferase (AST), Nonalcoholic fatty liver disease (NAFLD), Long non-coding RNAs (lncRNAs), Gene Expression Omnibus (GEO), Tubulin Alpha 1c (Tuba1c), CKLF Like MARVEL Transmembrane Domain-Containing 7 (Cmtm7), ABI Family Member 3-Binding Protein (Abi3bp), Activity-Regulated Cytoskeleton-Associated Protein (Arc), Chondroitin Sulfate Synthase 1 (Chsy1), Lipopolysaccharide-Induced TNF Factor (Litaf), Docking Protein 1 (Dok1), C-X-C Motif Chemokine Ligand 5 (Cxcl5), Formin 2 (Fmn2), Small Nuclear Ribonucleoprotein Polypeptides B And B1 (Snrpb), Von Willebrand factor (Vwf), Cold-inducible RNA-binding protein (Cirbp), RNA-binding Motif Protein 3 (Rbm3), Cholesterol 25-Hydroxylase (Ch25h), Neuronal Differentiation 2 (Neurod2), Platelet-type phosphofructokinase (Pfkp), 3-Hydroxyacyl-CoA Dehydratase 2 (Hacd2), Solute Carrier Family 39 Member 1 (Slc39a1), Ferritin light polypeptide 1 (Ftl1), Phytanoyl-CoA Dioxygenase Domain-Containing 1 (Phyhd1), Deoxyribonuclease 1-like 2 (Dnase1.2), Activin A receptor type I (Acvr1), Chromogranin A (Chga), PDZ domain-containing protein 8 (Pdzd8), Homeostatic Iron Regulator (Hfe), Ribosomal protein L 10 (Rp110), Melanocortin-4 receptor (Mc4r), Signal recognition particle 9 (Srp9), low density lipoprotein receptor-related protein 8 (Lrp8), piccolo (Pclo), calsyntenin 3 (Clstn3), purine-rich element-binding protein A (Pura), MAPK8 Mitogen-activated protein kinase 8 (Map8), Brain-derived Neurotrophic Factor (Bdnf), 3-beta-glucuronosyltransferase 1 (B3galt1), ATCAY Kinesin Light Chain-Interacting Caytaxin (Atcay), Cytoplasmic linker-associated protein 2 (Clasp2), Fragments per kilobase of transcript per million mapped reads (FPKM), Gene ontology (GO), Database for Annotation, Visualization and Integrated Discovery (DAVID), Ras-extracellular signal-regulated kinase (ERK), α-amino-3-hydroxy-5-methyl-4-isoxazolepropionic acid (AMPA), N-methyl-D-aspartate (NMDA).
